# Creativity and modelling the measurement process of the Higgs self-coupling at the LHC and HL-LHC

**DOI:** 10.1007/s11229-021-03317-y

**Published:** 2021-08-07

**Authors:** Sophie Ritson

**Affiliations:** University of Sydney, Sydney, Australia

**Keywords:** Creativity, Measurement, Experiment

## Abstract

This paper provides an account of the nature of creativity in high-energy physics experiments through an integrated historical and philosophical study of the current and planned attempts to measure the self-coupling of the Higgs boson by two experimental collaborations (ATLAS and CMS) at the Large Hadron Collider (LHC) and the planned High Luminosity Large Hadron Collider (HL-LHC). A notion of creativity is first identified broadly as an increase in the epistemic value of a measurement outcome from an unexpected transformation, and narrowly as a condition for knowledge of the measurement of the self-coupling of the Higgs. Drawing upon Tal’s model-based epistemology of measurement (2012) this paper shows how without change to ‘readings’ (or ‘instrument indicators’) a transformation to the model of the measurement process can increase the epistemic value of the measurement outcome. Such transformations are attributed to the creativity of the experimental collaboration. Creativity, in this context, is both a product, a creative and improved model, and the distributed collaborative process of transformation to the model of the measurement process. For the case of the planned measurements at the HL-LHC, where models of the measurement process perform the epistemic function of prediction, creativity is included in the models of the measurement process, both as projected quantified creativity and as an assumed property of the future collaborations.

## Introduction

The Large Hadron Collider (LHC) is one of the largest and most complex experiments ever built, consisting of a 27 km ring in which proton bunches are accelerated and made to collide in bunches in four detectors. Each of these detectors was independently built and is run by a large experimental collaboration: the ALICE, ATLAS, CMS, and LHCb experimental collaborations. ATLAS and CMS are multi-purpose detectors that were designed to understand the origin of electroweak symmetry breaking, to search for physics beyond the standard model, and to perform precision measurements of processes within and beyond the standard model (ATLAS, 2003; CMS, 2002).^1^

In 2012, ATLAS and CMS both announced that they had seen evidence for a boson and measured its mass at approximately 125 GeV. This was significant evidence to suggest that ATLAS and CMS had determined the unpredicted mass of—and discovered the long sought, last to be confirmed, prediction of the Standard model (SM)—the Higgs boson (ATLAS, 2012; CMS, 2012). Confirmation of the existence of the Higgs is widely considered to complete the SM, the theoretical outline of the properties of and interactions between high-energy particles. However, whilst the measurement of the mass, and subsequent measurement of the spin of the boson and various couplings, indicates that it is very likely to be the Higgs predicted by the SM,^2^ more measurements are required to see if the Higgs does play the role in the SM that is predicted. One such measurement is the measurement of the interaction of the Higgs with itself, or the self-coupling of the Higgs. The measurement also has potential for new physics: should the measurement contradict the SM prediction, it would indicate non-SM contributions and could provide an indirect path to new physics.^3^ The measurement of the self-coupling of the Higgs has been attempted at both ATLAS and CMS at the LHC and is planned to be attempted with the approved upgrade to the LHC, the High Luminosity LHC (HL-LHC) (ATLAS, 2018a; CMS, 2018a). Whilst the HL-LHC is not scheduled to begin collecting data until 2026, preparation for the European Strategy Meeting in 2019 resulted in high-energy physicists from within ATLAS and CMS, together with some input from theorists, performing studies for the planned HL-LHC measurements (CERN, 2018).

This paper introduces an understanding of the nature of creativity that is grounded in experimental practices via a two-part case study of attempts to measure the self-coupling of the Higgs at the LHC and the studies for the measurement at the HL-LHC. The paper begins with a notion of creativity identified during interviews with experimental physicists. This notion of creativity is perhaps not expressively ‘grandiose’, and certainly diverges from any understanding of creativity linked to theoretical hypothesis formation, or to individual genius. It is, nonetheless, significant in that it offers a starting point to gain insight into creativity as an epistemic concept in the context of collaborative measurements.

Semi-structured interviews were conducted with experimentalists who worked at different levels in CMS and ATLAS experiments (those working on the analysis, and coordinators)^4^ on the measurement either at the LHC or on the studies for the measurement at the HL-LHC. All participants have been anonymised in this paper and assigned unrelated surname pseudonyms. During these interviews, I was surprised that the interviewees used the concept of creativity explicitly, and in a particular way, as one of the conditions needed for a measurement of the self-coupling of the Higgs, and more broadly in their descriptions of the experimental collaborations. In addition to analysing the interviews with physicists I also analyse publications from the ATLAS and CMS collaborations to grasp the particular notion of creativity. This two-fold approach is necessary to move beyond the rhetorical outline of creativity offered by the physicists, to analyse the epistemic practices that the interview subjects associate with creativity.^5^

For the case study, I draw upon Tal’s model-based epistemology of measurement which focuses on the role of models of measurement processes. There are two classes of models of the measurement process constructed for two different experimental apparatuses: a class of models for the LHC, and a class of models for the HL-LHC. The two classes of models of the measurement processes also differ in that they are constructed for distinct epistemic functions: specification of the content of measurement outcomes and prediction of measurement outcomes. Each of these functions is examined from the context of the notion that ‘creativity’ is required for a measurement of the self-coupling of the Higgs. It is also noteworthy that for both of the classes of models, the model of the measurement may not, most likely will not, represent a measurement process that will result in a measurement of the self-coupling of the Higgs. For the LHC models the epistemic aim is to assign upper and lower limits of the Higgs self-coupling. For the HL-LHC models, the models are not intended to represent a realised measurement process: instead, the epistemic aim is to gain knowledge of the capacity of the measurement processes for measurement outcomes.^6^

The paper will proceed as follows. I will first outline different treatments of the concept of creativity, highlighting the ambiguity of the concept. I argue that one source of this ambiguity is a dynamic tension in the concept of creativity. For clarity I will describe conceptually how the measurement of the self-coupling of the Higgs can be achieved. Following this, I will outline the difficulties of completing this measurement at the LHC in practice, including the perceived need for creativity. This also serves as an introduction to the physicist’s conception of creativity in the case study, broadly as an increase in the epistemic value of a result from an unexpected transformation, and narrowly as a condition for a measurement of the self-coupling of the Higgs. I then interrogate this notion, first in the context of the measurements at the LHC, and second in the context of the planned measurements at the HL-LHC. In the first part of the case study, I show how a transformation to the model of the measurement process can transform the measurement outcome, without change to the instrument indicators: a transformation attributed to creativity. Creativity, here, is both the product, a creative model, and the process of transformation to the model of the measurement process. Creativity is both identified and quantified through comparisons to transformations from expected contributions. However, some in the collaboration, highlighting that the value of novel contributions is non-trivial, contest certain transformations to the model. I show that this is an example of the dynamic tension in creativity. In the second part of the case study, I show how models of measurement processes can have the epistemic function of prediction. In these models, quantified creativity from the LHC models is projected and tightly constrained such that the projections are meaningful. The modelling activity aims to make predictions of future measurement processes. These predictions provide knowledge of a future that will never occur, as the material conditions of that future will determine the subsequent transformations; instead, knowledge of capacity for measurement outcomes is obtained.

## Creativity

Creativity, where defined,^7^ is very often defined as having two necessary conditions: novelty and value (or utility).^8^ Across the various attempts—coming from history, philosophy, psychology, and sociology—to define creativity, or outline conditions for creativity, creativity is assembled in various ways. There are significant departures concerning the sufficiency of novelty and value, and various interpretations of novelty, value, and the relationship between novelty and value.^9^ Four points of contention are as follows.

*Is creativity a property of an individual, or a group?* In addition, *is creativity a property of a process, or a product?* Here the concern is what is the vehicle for creativity. Much of the literature considers that it is products which are relevant for the study of creativity, including ideas and material objects such as pieces of artwork or scientific hypotheses (see, for example, (Boden, 1994a)). Other literature asserts that because it is conceivable to have a creative process that does not result in a creative product, it is the process that is relevant, such as group brain storming sessions (see, for example, (Lubbart, 1999)). Certain accounts of creativity, coming from social epistemology or history, have argued that it is difficult to link creativity with individuals. These accounts argue that from a detailed historical or sociological perspective creativity is ‘distributed’ across multiple individuals, and that material and social arrangements are important for understanding conditions for creativity (Currie, 2018a, 2018b; Miettinen, 2006, p. 173; Pinch, 2015, p. 142). Finally, some literature, often psychology literature, considers creativity as a property of individuals, such as where creativity is deemed a particular psychological state (see, for example, (Lumsden, 1998)). Note that all three vehicles are used commonly: ‘Ada Lovelace was very creative’ (property of an individual); ‘*Nanette* was a very creative piece of stand-up comedy’ (property of a product); ‘John and Paul collaborated creatively together’ (property of a process and a group, and sorry, Ringo and George!).

*What level or ‘kind’ of value is sufficient?* In addition, *what level or ‘kind’ of novelty is sufficient?* These discussions also run along similar lines, so I include them both here. Authors argue that in order to identify creativity in any context such as art or science, there ought to be a threshold for the level of value, or the level of ‘newness’, or both, otherwise there is the risk that creativity will become trivialised and the concept rendered meaningless. Boden (2004), for example, identifies three different categories of novelty: unfamiliar, unexpected, and apparently impossible. The first is something made from unfamiliar combinations of familiar ideas; the second is something previously unthought, that is consistent with the conventions or rules of a conceptual space^10^; and the final is something that is new which could not have, with respect to the conceptual space, occurred before (Boden, 2004, pp. 3–6). Alternatively, other authors argue that novelty and value are insufficient on their own, and that something else must be required, such as ‘intentionality’ or a defined relationship between novelty and value (Gaut, 2010, p. 1039; Klausen, 2010).

As is clear from the divergences in the literature, creativity is an ambiguous concept that permits multiple understandings. I argue that one source of this ambiguity comes from the tension in the concept of creativity, which is formed of two concepts (novelty and value) that are in direct conflict. To value is inherently conservative, as it takes place within an existing framework, or conceptual space (to use Boden’s terminology). When anything is valued, it is because it adheres to, corresponds with, or is complementary to an existing conceptual framework. However, the production of novelty is the opposite: it requires change and transformation. As Klausen outlines, “creativity is about breaking with norms and practices, doing something unexpected or unpredictable, but still meeting certain—albeit more liberal—constraints” (Klausen, 2010, p. 349). Thus, a creative process is one that simultaneously transforms one conceptual space whilst remaining consistent with another, higher order or alternative conceptual space. A creative process or product requires conservative change. Note that I do not offer a definition here; instead, it is a working understanding of the concept of creativity derived from the inherent tension in the standard necessary conditions for creativity.

## The Higgs self-coupling measurement and the need for ‘creativity’

**Fig. 1:**
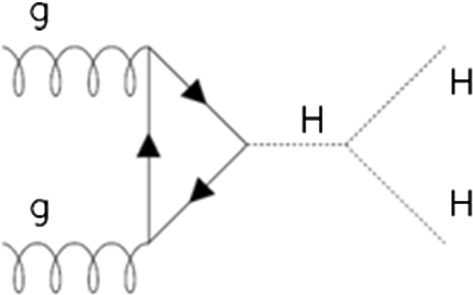
Source: (Blondel et al., 2002)

A measurement of the Higgs trilinear^11^ self-coupling (*λ*_HHH_) is considered achievable from an ‘observation’ of Higgs boson pair production. Figure 1 shows a Feynman diagram of Higgs boson pair production via Higgs self–coupling via gluon fusion.

The strength of the trilinear coupling influences the production rate of Higgs pairs in the SM, because *λ*_hhh_ is related to the probability of the Higgs to ‘decay’ into a pair of Higgs. The rate for this decay, and therefore the production rate, is higher if λ is higher, or lower if λ is lower.^12^ The Higgs potential can be accessed experimentally through the measurement of the cross section for Higgs-pair production (where a measurement of the cross section (σ_pp_ → HH) is a measurement of the yield of all processes that produce two Higgs, including the Higgs self-coupling). The Higgs sector may be different from that which the SM predicts, which can lead to a measurement of a different rate of HH production, yielding a different value of λ_HHH_. It is then possible to compare the experimentally determined λ_HHH_ with a theoretical prediction for the SM self-coupling* λ*_HHH(SM)_. If λ_HHH_/_λHHH(SM)_ is compatible with 1, this constitutes a measurement of the SM self-coupling (Blondel et al., 2002).

In practice there are several challenges that need to be overcome, some of which are generic to LHC measurements and some of which are particular to the measurement of the self-coupling of the Higgs at the LHC. The generic challenges stem from the impossibility of the detectors to ‘observe’ directly, or interact with, the short-lived particles, such as Higgs bosons, created by the collisions. Instead, the multi-purpose detectors at the LHC (ATLAS and CMS) are optimised for the decay products. For example, the initial evidence found for the Higgs was a measurement of an excess of two of its possible decays, two photons (γγ) and ZZ^13^ (ATLAS, 2012; CMS, 2012). However, the decay products are also not directly observable, although they do interact with the detector to produce electronic signals (instrument indicators). The decay products relevant to the search or measurement performed must be inferred from the satisfaction of specific data selection criteria on the instrument indicators. However, these criteria will also select events that are indistinguishable from the relevant decay events (background) and will reject events that are relevant. Therefore, both the number of background events and the efficiency of the selections need to be estimated in order to make corrections. In addition, the detectors are unable to achieve 100% coverage, so the acceptance rate must be estimated, and detector resolution effects can have a small impact, corrected either by a process called unfolding or by comparison to simulations. Each of these transformations has an associated uncertainty that also needs to be calculated (Beauchemin, 2017).^14^ Finally, the complexity of ATLAS and CMS experiments is such that the experiments require collaboration between the experimental collaboration members, as it is practically impossible for an individual to complete an analysis. Very often, it is also the case that collaboration with theorists or phenomenologists provides an advantage. For example, collaboration with theorists or phenomenologists can assist with calculating expected production rates.

On top of all these generic requirements to complete a measurement, the self-coupling presents many additional challenges. The rate of diHiggs production, predicted by the SM, is very small, so the number of candidate events produced in colliders is very small, requiring very small uncertainties to achieve sensitivity to the measurement objectives. By way of comparison, single Higgs production occurs by a factor ~ 1000 more often than diHiggs production. For the LHC there are five viable decay channels for diHiggs: bbbb, bbWW, bbττ, bbγγ, WWγγ. Each decay channel has its own specific background difficulties. In addition, the theoretical prediction for λ_HHH(SM)_ also has an associated ‘uncertainty’, so in order to compare the theoretically derived prediction, the experimental precision must match (Jézéquel, 2017).

Given the difficult experimental conditions, physicists in both the ATLAS and CMS collaborations also highlighted the need for “creativity” to complete the measurement, as will be explored in the following sections. One interview participant, Barrie, indicated that they were optimistic about the potential for creativity.

Interviewer: And this optimism, that if people are given the space and the time that they will be able to be creative?

Barrie: Yeah, I mean we’ve seen that. If you look at what the projections [were] for the LHC and what we actually did, we discovered the Higgs with half the energy and very little data in one of the more tricky areas.

Barrie not only outlines their source of optimism (past performance), they also identify what they have observed as creativity. Here, Barrie contrasts the physics prospects studies, completed before the construction of the LHC and which gave an indication of the capacity of the LHC to discover the then yet to be discovered Higgs boson, with how the discovery was obtained by the collaboration in practice. Barrie observed the improvement in the performance of the collaborations, which was able to measure the mass of the Higgs boson with less data and less energy, as creativity. Embedded in Barrie’s optimism for future creativity is a historically situated notion of creativity, identified through the comparison of the improvement of the collaborations against the increasing amount of data, or accessible energy.

Another interview participant, Rooney, also identifies creativity through comparison.

Interviewer: And do you think there's a role for creativity, for the members of the collaboration? [Rooney: Yeah yeah] What do you think this role is? Or what do you think creativity is in this sort of context?

Rooney: Well I mean you have the creativity… essentially you have an experiment which was planned uh twenty years ago, [and] you had optimized essentially for finding the Higgs mainly. And now we are covering more and more different physics scenarios which were never on the on the radar when the experiment was designed. This kind of proves that people are always coming up to find solutions, to use the same apparatus which essentially was designed for something else, to use it in new ways to get new results. For example, one of the first analysis we have now released with the run 2 data is for these long-lived particles. The detectors have a certain capacity to measure time, and in a certain window. Initially the main reason for that was because we have these collisions every 25 nanoseconds. Now people are coming up with methods to use this capacity of the detector to see if a particle was moving slow up or was not moving in the direct path but made a kink somewhere, which is a creative solution to use an existing detector for a new purpose.

In this case, the experimental apparatus is being used for measurements in a way for which it was not designed. Rooney compares the expectations of the experimental apparatus against the contemporary performance of the collaborations, attributing the difference to creativity. Here we can begin to understand why creativity was expressed as a condition for knowledge formation, in the context of the considerable difficulties of measuring the self-coupling of the Higgs in practice: the predicted low production rate and difficult backgrounds result in very little data, requiring collaborations to model the measurement process such that more can be done with the available data. Creativity, following this understanding, is linked to novel transformations and in this particular case is a condition for a successful measurement outcome.

## Models of the measurement process for the LHC

The first part of this case study considers the attempts to measure the self-coupling of the Higgs boson at the LHC, attempts that face a number of challenges in practice. One of the most significant challenges is the low diHiggs production rate predicted by the SM, so low that it is considered impossible for either ATLAS or CMS to measure the SM self-coupling of the Higgs at the LHC. Not only can they not ‘discover’ (i.e. produce a 5-sigma result), but it is considered impossible to generate a 3-sigma result, or ‘evidence for’, the SM Higgs self-coupling^15^ either from a single channel or from combining channels. Despite this there are numerous published results from the data collected at $\sqrt{s}=8 TeV$ and at $\sqrt{s}=13 TeV$ at the LHC. Researchers interviewed cited multiple motivations for these analyses, including the generation of improvements in understanding within the collaboration of analysis techniques in relation to the data. A further motivation was broadly the potential to “get lucky”. The potential to “get lucky” is derived by the physicists from theoretical predictions from BSM physics. As the ATLAS collaboration states, and as is typical to state in these publications, “many [BSM] models predict cross sections for Higgs boson pair production that are significantly greater than the SM prediction” (ATLAS, 2018b, p. 1). Which is to say that if the self-coupling of the Higgs is as predicted by one of the various BSM models, more events will be produced to the point where a result could be measured in one of the decay channels at the LHC. Before exploring the measurement attempts at the LHC, I will first briefly outline Tal’s model-based account for the production of knowledge in measurement.

### Models of measurement processes

Tal’s model-based epistemology of measurement draws attention to the epistemic function of models of measurement processes (Tal, 2012, 2013, 2017c). Tal argues that there are two kinds of measurement outputs: ‘instrument indications’, or ‘readings’, and ‘measurement outcomes’, or ‘results’, where the measurement outcomes are knowledge claims, and rest on the ability to infer from instrument indications. For Tal,

the term “model” denotes an abstract and local representation of a measurement process, that is, a system composed of a measuring instrument, objects or events to be measured, the environment (including human operators), secondary instruments and reference standards, the time-evolution of these components, and their various interactions with each other. (Tal, 2017b)

Tal has, and other authors of model-based accounts^16^ have, stressed that the ability to infer from instrument indications to measurement outcomes is “nontrivial” and “depends on a host of theoretical and statistical assumptions about the object being measured, the instrument, and the calibration process”. Tal proposes that models that represent the measurement process are necessary preconditions for the possibility of inferring measurement outcomes from instrument indications. Models of the measurment process are therefore “crucial for determining the content of measurement outcomes” (Tal, 2017b). One of the key contributions of this account is to note that it is the epistemic function of the model of the measurement process to relate instrument indications to the measurement outcome, and to represent the final states of the process (Tal, 2012, p. 19).

This paper follows the insights of this approach in the context of high-energy physics measurements. However, instead of a focus on standardisation and explanation of the stability of measurement, I will now examine creativity in the context of transformations to the models of measurement process of the self-coupling of the Higgs.

### Uncertainty and quantified creativity

In this part of the case study, I focus on HH → bbbb in ATLAS as an example of one of the ways in which the measurement process is modelled.^17^ In this channel, each Higgs boson decays into two b-quarks. The channel is considered interesting for the purposes of the self-coupling measurement as the number of events is predicted to be higher than for other channels; however, it suffers from a large number of events that also produce four b-jets (a large background).

There are two publications of interest, both drawing upon data from the 2015 run at 13 TeV (with the same titles) (ATLAS, 2016d, 2018b). Two analyses from data taken from the same run is unusual given that it takes a great deal of time and effort for a group to complete an analysis and get it approved for publication within the collaboration. Due to this, it is common practice to wait until there is a significant increase in the amount of data before attempting a publication, and conducting and publishing an analysis can easily take three or more years from start to finish. However, in the 2018 paper, a result from the 2015 run is published, as well as a new result from the 2016 run. Given that the instrument indicators did not change between the 2018 paper and the 2016 paper, for results from the 2015 run, the 2018 paper highlights that something else must have changed to make the results worthy of publication. The result achieved from the 2016 run is still very far from the SM rate and puts a limit on the result to be less than 13 times the SM rate (ATLAS, 2018a, 2018b). This result is referenced in a CMS paper as giving the “tightest upper limit on SM non-resonant HH production set so far by LHC searches, obtained with the search in the bbbb channel” (CMS, 2018d). This change comes from improvements in the assessments of systematic uncertainty.

Systematic uncertainty is a concept that requires further clarification. Beauchemin, a practitioner of measurement in the ATLAS collaboration, outlines systematic uncertainty as the quantification of the “potential variability of an experimental outcome due to all the anticipated variations of the many theoretical assumptions and the auxiliary data needed to obtain the result” (2015, p. 282). Thus, systematic uncertainties specify to what extent a measurement outcome “could vary if the procedure was performed in different but equivalent conditions” (p. 299). Variations can arise from statistical fluctuations of the phenomenon measured, fluctuations in the measurement apparatus, and “indeterminacies in the multiple assumptions, models and theories used” (p. 299). For example, for the evaluation of jet uncertainty in the HH to bbbb 2018 analysis, one contributing factor to the systematic uncertainty is that there are several competing theoretical models of the interactions between hadrons and the material in the calorimeter in the detector. As the choice of theoretical model affects the measurement outcome, the extent to which the measurement outcome changes with different choices is calculated and included in the overall model of the measurement process through the evaluation of the systematic uncertainties [(ATLAS, 2015a) as cited in (ATLAS, 2018b)].^18^

In the paper, the improvements outlined come from two complementary analyses, each of which employs a novel technique in the reconstruction of the Higgs bosons (ATLAS, 2018b, p. 2). B-tagging is the process used to describe the identification of the jets produced by b-quarks, thereby identifying candidate b-quarks from which the presence of each of the two Higgs bosons is inferred. In the case of the diHiggs, four b-jets are used to infer the presence of two candidate Higgs particles. One analysis (the resolved analysis) used is where the Lorentz boost of the Higgs bosons is low enough that the four b-jets can be reconstructed. The second (the boosted analysis) is where the Lorenz boost of the Higgs bosons is higher, preventing the separate resolution of the b-jets in the detector, and the presence of the Higgs candidate is inferred using charged particle tracks to build smaller radius jets. An improved algorithm is used in the resolved analysis that helps identify which b-jet came from which Higgs boson candidate and an additional signal-rich sample is used in the boosted analysis (ATLAS, 2018b). Identifying which candidate intermediate Higgs boson is associated with which pair of b-jets results in an improvement in the uncertainty of the selections of diHiggs events against the background. Table 1 below reconstructs the improvement in the systematic uncertainties from the published result in 2016 with 3.2 fb^−1^ of data and the results published in 2018 with 36.1 fb^−1^ of data.

**Table 1:** Systematic relative uncertainties (expressed in percentage yield) (Atlas, 2016d, 2018b)

Source	2015 Run (ATLAS, 2016d)	2015 Run (ATLAS, 2018b)	2016 Run (ATLAS, 2018b)
Luminosity	5	2,1	2,2
Jet Energy Resolution	2	–	–
Jet Energy Scale	12	–	–
Jet Energy	–	7,1	6,4
b-tagging	18	12	12
Theoretical	9	7,2	7,2
Total	24	16	16

As Tal (2012) has outlined, the crucial function of a model of the measurement process is to specify what the physical and symbolic aspects of the instruments are measuring and to determine the content of measurement outcomes. Here we see an example of a transformation to the model of measurement process, in that there is a change in the representation of what the measurement process is measuring, resulting in a transformation to the measurement outcome. An example of what Tal has described is a case of the “same indications produced by the same measurement process used to establish different measurement outcomes depending on how the measurement process is modelled” (2015). The 2016 iteration of the measurement process has an improved measurement outcome, despite no change to the instrument indicators. This highlights that the evaluation of systematic uncertainties is also modelling activity, which incorporates multiple representations of the measurement process to determine the epistemic value or validity (or, as Staley (2018) has argued, the robustness) of measurement results. Therefore, the transformation, depicted in Table 1, of the 2015 data is a transformation in knowledge of how the measurement process is modelled, a transformation that, in this case, results in a measurement outcome with greater epistemic value. This increase in epistemic value results from a higher level of specification as to what the physical and symbolic aspects of the instruments are measuring, for the purposes of establishing liminal knowledge of the potential range of measurement outcomes.

One interview subject, Cohen, who was one of the many who collaborated to produce this analysis, attributed this transformation to creativity:

How we change the analysis, I think that’s up to the creativity of the scientists … What we’re seeing is an analysis that’s improving at a really great rate, improving much more than just adding more data … So, there’s just technological improvements in all aspects of the analysis and our ability to understand our data so that our uncertainty about our data reduces, what we call systematic uncertainty, or how well we understand the modelling of the processes. Not just how much data we have, the statistical uncertainty, but really how well we can model it.

Cohen here draws a comparison between improvements in systematic uncertainties and improvements in statistics (the amount of data). This also recalls the concept of creativity invoked by Barrie and Rooney in their reflections in Sect. 3. Through the comparison of improvement due increases in the available data and systematic uncertainties, we can see how it is possible to identify and measure creative contributions. This comparison process generates an improvement not attributed to an expected increase in data but rather an improvement attributed to the collaborative effort, effectively a quantification of creativity as a process. Many of the ambiguities in understanding creativity are present in this notion of creativity. Creativity is identified through a comparison of improvements in the measurement outcome, between those attributed to increases in data and those attributed to other improvements such as a decrease in the systematic uncertainties from a transformation in the model of the measurement process. This notion also lends itself to considerations of creativity as a product: unexpected improvements to the model of the measurement process. It also permits even very small improvements to be identifiers of creativity, as long as there is a net positive change across a comparison (all other things being equal).^19^ This notion runs counter to other previously discussed notions of creativity, as it removes any requirement for a necessary ‘level’ of, or threshold for, novelty.

However, the absence of a threshold for novelty is only part of the notion of creativity here. The collaborative context in which this example of creativity is embedded is also important. One interview participant, Reed, outlined the processes by which improvements are achieved:

we go into each of the subtasks in the analysis techniques and we try to improve each of them and then, even if you improve by 10% – everywhere by 10%, [the analysis] improves in the end by quite a significant fraction, so that’s the way we do. I mean of course we try to find – something maybe new and novel, but not always it works. Sometimes you just try to improve what’s existing by adding something new. Maybe not a brand-new technique but it is something new.

Here Reed emphasises the lack of need for a threshold for novelty when the analysis is broken down into parts, with multiple ongoing contributions. This is also reflected in Table 1 where there are improvements across each of the listed systematic uncertainties, each of which contributes to the improvement in the total systematic uncertainty and the epistemic value placed on the result. The dual aspects of creativity as a dynamic and distributed process and as a quantifiable product are also evident from this account in the case of multiple contributions (distinct products), combined from multiple sources (a dynamic process drawing on distributed distinct products). The potential net effect of this distributed process is something that may generate a sufficient change in the epistemic value of the measurement outcome to be recognised within the collaborations (in the case of the example above, as being sufficiently novel to warrant the publication of an improved analysis). In this context, creativity is a distributed process that transforms the model of the measurement process such that each new iteration increases in epistemic value.

### Uncertainty and the tension in creativity

The identification of creativity from the perspective of approved and published analyses, however, obscures some of the complexities of how measurement knowledge is produced in the experimental collaborations. In particular, it obscures how models of measurement processes are constructed and recognised within the collaborations. One issue of concern at the time of the interviews and for the measurement of the self-coupling of the Higgs was the use of machine learning (ML), such as boosted decision trees (BDT) or deep neural networks in efforts to ‘do more with the data’ or exceed expectations. Broadly, ML techniques are utilised when making inferences from data, where the pathway to the inference is not explicitly programmed. There is a great deal of diversity in ML techniques^20^; however, as Wheeler has argued, what sets ML apart is that it “aims to circumvent the requirement to explicitly set modeling assumptions prior to drawing meaningful inferences or, when unavoidable, seeks to design algorithms that learn on their own what modeling assumptions are best to select” (Wheeler, 2017, p. 323). Neural networks have historically been successful in the experiments, for example for b-jet identification, and the associated processes for determining the systematic uncertainties are now both well understood and established within the collaborations (ATLAS, 2016a). Interview participants reported that, more recently, new ML techniques have been imported from industry for use in the analysis, often from the tech giants.

Recently historians and philosophers of science have begun to question whether ML is a black boxed process that introduces ‘epistemic opacity’.^21^ Relevant for this paper is what role such considerations play in discussions of experimental reasoning both in the interviews conducted and in the published papers of some physicists. Here I compare two different perspectives on the potential of ML from two interview participants working on the Higgs self-coupling measurement:

they’re beginning to use more and more machine learning as we get more and more comfortable with the data and we really believe that we understand it. You see more and more use of machine learning and that really does help push the boundaries even more. So that’s another one of those places where we’ll see the analysis improve faster than just adding more data. (Cohen)

the drawback is that it’s easier to implement machine learning than to look at [the] more complicated problem, which is developing a new algorithm ... I think the work of physicists ... so machine learning is a tool but you should ... go beyond. Our added value is to understand the results, understand if they are reasonable. (Birkin)

Whilst Cohen links ML to deeper understanding, Birkin expresses resistance to the recent use of ML, and instead emphasises how physicists can increase the epistemic value of their results. If we consider the notion of creativity explored so far, as processes and products linked to increases in the epistemic value of results through unexpected transformations, Cohen links ML to the potential for creativity, whereas Birkin emphasises the potential of physicists for creativity. Key to this divergence are different perspectives on how the model of the measurement process should be developed and transformed. For Cohen, ML simultaneously deepens the understanding of the model of the measurement process for the epistemic agent and improves the measurement outcome. Birkin is sceptical of the value of including ML in the measurement process. Instead, Birkin emphasises the positive potential of the physicist, as the epistemic agent, to introduce novel contributions to the model of the measurement processes.

Chang et al. have also recently observed similar sceptical arguments amongst high-energy experimental physicists:

A common argument against using machine learning for physical applications is that they function as a black box: send in some data and out comes a number. … a physicist often wants to understand what aspect of the input data yields the discriminating power, in order to learn/confirm the underlying physics or to account for their systematics. (Chang et al., 2018, p. 1)

In the context of measurement, the scepticism that Chang et al. outline is based on the potential for ML to obscure aspects of the measurement process, thereby introducing obstacles to representing the measurement processes and determining the associated systematic uncertainties. Key to this conflict is the questions of whether ML can be creative in the sense identified in Sect. 4.2 (both process and product); that is whether ML can result in improved systematic uncertainties, where systematic uncertainty is a quantification of the level of specification of what the measurement process measures. The notion of creativity invoked as a requirement for a measurement of the self-coupling of the Higgs was not only associated with novel techniques that result in transformation to the measurement outcome; it was also the case that the transformation resulted in an increase in the epistemic value of the measurement outcome. The recognition of an improvement in the measurement outcome is dependent on the recognition of the trustworthiness of an improvement in the model of the measurement process. This latter condition is crucial to understanding creativity as a condition for measurement in this context.

Supporters of ML do not contest that novel techniques should not result in a decreased understanding of the model of the measurement process. Reed, who was working directly on how ML might contribute to the measurement of the self-coupling of the Higgs in CMS, emphasised the openness of the processes used to check ML and to what extent they differ from industry:

You cannot just take use the machine learning and then you say that’s what our final result is because that’s what this black box told us. It’s never like this. While normally, in the big companies, it’s not really needed because, normally it’s all aimed for humans, and then you see … if something doesn’t make sense, it doesn’t make sense, right? While, here, it’s much harder to understand, if you look at the result whether it’s true or not.

These discussions are often framed in terms of the importance of “physics intuition, where intuition is described as an understanding of the underlying physical and experimental processes” (Chang et al., 2018, p. 5). Reed here argues that the processes by which ML techniques are tested are such that black boxes are not introduced to the model of the measurement processes. Other supporters of ML have also claimed that ML can “expand physical intuition” (Larkoski et al., 2017, pp. 34–35) or that ML “is most efficient in tandem with physics intuition” (Chang et al., 2018, p. 5). In doing so, these supporters affirm the importance of transparency for the recognition of the value of novel techniques.^22^

Key to the discussion here is the relationship between a notion of creativity, understood as both a product and a process, and the epistemic function of the model of the measurement process. In the case of the LHC models of the Higgs self-coupling measurement process, there is consensus that alterations to the models of the measurement process should not prevent models from being able to perform their epistemic function. Instead, the source of conflict is whether certain ML tools should be recognised as sufficiently transparent, as this transparency is one key to the evaluation of systematic uncertainties and the recognition of the epistemic value of a measurement outcome (or the trustworthiness of the result). This focus on transparency highlights the tension in creativity, where change must be conservative. The source of this requirement, in this case, is that the epistemic function of the model is to specify what the instruments are measuring. In this context, in order for novel contributions to have epistemic value and be included in the models of the measurement process, they must be recognised within higher order conceptual spaces. The tension in creativity is played out in the distributed and iterative dynamics of collaborative creativity. Novelty is constrained to be transparent in the conceptual space in which value is determined, in this case in the evaluation of systematic uncertainty and the attribution of epistemic value. The epistemic function of the model of the measurement process is the conservative force, taken into consideration in the recognition of the value of novelty.

## The measurement process model for the HL-LHC

The upgraded LHC, the HL-LHC, is scheduled to be completed in 2026. Also to be upgraded are the detectors, including the ATLAS and CMS detectors.^23^ The HL-LHC is designed to deliver an increase in the integrated luminosity (the total collisions created) by a factor of ten compared to the LHC. Utilising this increase in data to measure the SM cross section (σ_pp_ → HH) by 2036 and the SM self-coupling, or otherwise to put constraints on the self-coupling of the Higgs, is often described as a “flagship” measurement for the HL-LHC as a measurement of the SM value is not predicted to be possible without the HL-LHC.

Tal (2017a) has drawn attention to the close relationship between measurement and prediction “which has thus far remained implicit in philosophical writings” (p. 44). Here we have a case of an attempt to generate a meaningful prediction of the capacity of a future measurement process (for measurement outcomes), a significant task undertaken over several years, published in a CERN Yellow Report.^24^ A draft version of the report was made publicly available late in 2018, authored by some members of the CMS and ATLAS collaborations, as well as some theorists and phenomenologists (CERN, 2018). The measurement process is modelled in the report using extrapolations from the current measurements processes, adjusted for the different conditions at the HL-LHC. However, the HL-LHC measurement is not intended to represent the measurement practices that will occur at the HL-LHC (for example, the algorithms used to analyse the data will be replaced by 2026).

**Fig. 2:**
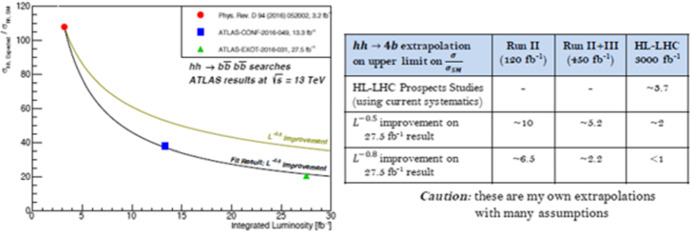
Source: (Kagan, 2018)

In Fig. 2 Kagan, from ATLAS, presents his extrapolations based on his analysis of the rate of improvement in the HH → bbbb from consecutive iterations of a LHC model against expected improvements due to an increase in data (or statistics).

These extrapolations are based on the same method to measure creativity identified in Sects. 3 and 4.2, however here the rate established is projected into the future (including for the HL-LHC model). If we take this projection as a projection of creativity, this assumes that creativity is an ongoing property of the ATLAS collaboration, identified by past processes and projected into the future. Beyond the assumptions that he is required to make, Kagan identifies one further difficulty when he states that it is “hard to indefinitely improve analysis … will we run into a ‘systematics wall’?” (2018). This highlights the difficulty of translating the rate of improvement, or the quantified creativity, from the LHC model to the HL-LHC model. Even when creativity is assumed to be a property of the future collaboration, other factors must be taken into consideration. For these prospect studies simply to assume historic rates of creativity, based on how the collaborations have worked in the past, would be to undermine the epistemic function of the model of the measurement process, as it would deny the changing relations between the material and epistemic conditions for the HL-LHC. The inclusion of projected creativity in the HL-LHC model is thus tightly constrained theoretically, experimentally, and practically.

**Fig. 3:**
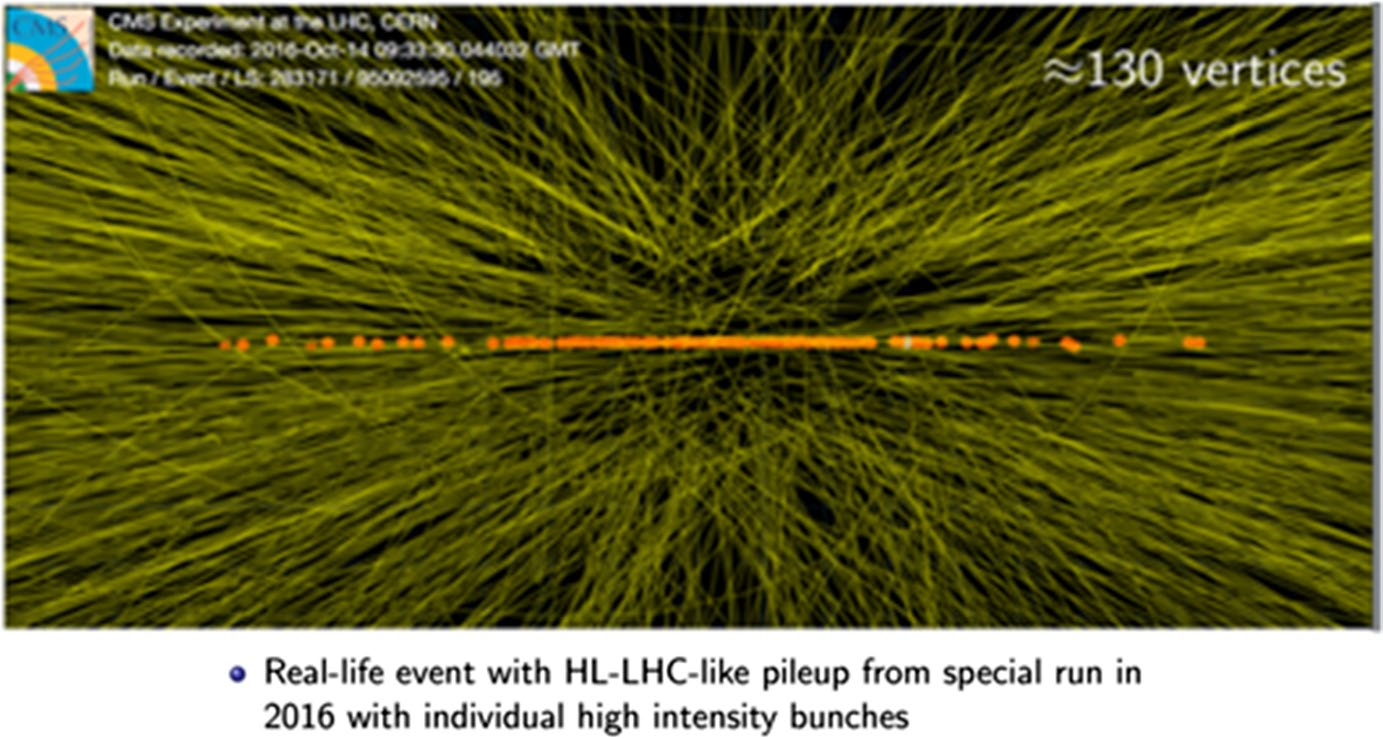
Source: (Bendavid, 2017)

There are several differences between the representational context of the LHC model of the measurement process and the HL-LHC model, which constrain the projections. A certain level of consistency with the LHC models from each of the separate channels is required so that the extrapolations from the LHC measurement models can be included in the HL-LHC model. The HL-LHC measurement is also modelled in the Yellow Report on a different scale: rather than only single channels, the measurement process is modelled based on combining all of the channels in both ATLAS and CMS. This requires very complex uncertainty calculations and a simplified model is presented in the Yellow Report (CERN, 2018, p. 102). A further challenge is that the conditions in the HL-LHC model are significantly different to those in the LHC model. Whilst there is the benefit of additional signal from the increased luminosity (and from combining the channels and ATLAS and CMS), some conditions are predicted to result in a decline in prospects. To achieve luminosity there will be 200–300 simultaneous collisions in each bunch crossing (known as pileup), and of those collisions at best one collision may be relevant. See Fig. 3 for an event from CMS in 2016 used to illustrate the pileup conditions at the HL-LHC.

For the Yellow Report, 200 simultaneous collisions are assumed. The result of the high pileup conditions is that experimental uncertainties are in some cases estimated to go up, despite the increased signal. The upgraded detectors have been designed to compensate for the high pileup conditions, but it was not feasible to produce full simulations of all channels of the yet to be built detectors for the Yellow Report (CERN, 2018). Accommodating each of these difficulties constrains the space for future creativity in the projections.

Returning to our example from the HL-LHC models, the measurement in the HH → bbbb channel in ATLAS is modelled according to the following assumptions (CERN, 2018, pp. 91–93). It is assumed that the performance achieved in 2018 can be achieved based on improvements from the 2018 analysis and that the improved detector will compensate for degradations due to pileup. We can see here that the projected creativity from the 2018 result is constrained by the material conditions of the HL-LHC. In order to establish an improvement against the 2018 results, an 8% increase in efficiency in b-tagging is attributed to the upgraded inner tracker of the ATLAS detector. Beyond this a minimum threshold of improvement is established by assuming that the analysis will be unchanged in terms of selection and statistical interpretation. This is explicitly identified as “pessimistic” (ATLAS, 2018a, p. 4) and “conservative” (CERN, 2018, p. 90) and improvements to future iterations of the model of the measurement process are expected due to novel techniques that will be established. Here we see simultaneously an assumption of no further creative contributions to the model, establishing a benchmark expectation for a measurement outcome, and also the expectation that future creative contributions to the model will occur, establishing a reach or potential of future iterations of the model.

Tal has drawn attention to the close relationship between measurement and prediction as it concerns calibration (2017a). Here, the model is not intended to represent a measurement process that will occur; instead, the model is an instrument for prediction of potential measurement outcomes. The epistemic function of the model is to provide a meaningful model of the capacity of the future measurement process for measurement outcomes, both in terms of a threshold expectation of the measurement outcome, and further potential for measurement outcomes with greater epistemic value. A meaningful model of the capacity of a measurement process is both comparable to current and future models, and is constrained by assumed material and epistemic conditions of the future.

Despite the difficulties in projecting creativity, spaces for future creativity were included in the HL-LHC model. As Lee, one of the co-ordinators involved in producing the prospect studies for the measurement of the self-coupling of the Higgs for the CERN Yellow Report, described it:

You maybe don’t know exactly what will be your precision at that time but what you can show is how your sensitivity of your measurement will change if you improve with respect to what you have today, if you improve it by 10, 20, 30, 40% - where it brings you. And, actually, we already agree that this is how we would like some of the results to be presented in this report. So that this report is not just a picture of the ultimate physics reach of the HL-LHC … it shows in many of the cases where more work is needed so it improves your sensitivity … it motivates the people and gives them the clear picture of where additional work in the future is needed to be done.

This implicitly includes future epistemic agents in the model. The source of improvements is outlined more generally in that a capacity for improvement is identified through the location of a space where it is possible. However, the precise nature of the improvements to the model of the measurement process is left to the future collaborations, which will need to construct further iterations of the model sensitive to material and epistemic conditions such as the designed but not yet built detectors.

There are two examples included in the draft Yellow Report for the measurement of the self-coupling of the Higgs, including ‘Prospects for bbγγ: Bayesian optimisation and BDT’ (CERN, 2018, pp. 106–109). This section is authored by Alves, Ghosh, and Sinha, phenomenologists who are not part of the collaborations (though the section is included due to an assessment of the section’s potential by the ATLAS and CMS Yellow Report authors). The phenomenologists offer a modified model of the measurement process in the bbγγ at the 14 TeV LHC that is not specific to either detector. It would therefore require further transformations to provide a measurement outcome with the associated uncertainties relevant to the detector used. The authors argue that their study shows that the BDT is better able to discriminate between signal and background due to an improvement in identifying correlations between the kinematic features of the signal and backgrounds, and is based on the change in signal significances (in standard deviations). The comparison the authors draw is in presented in Table 2.

**Table 2:** Signal significance for cut and count and BDT for 0, 10 and 20% systematics

Systematics %	Cut-and-count	BDT
10	2,34	3,88
**20**	**1,93**	**3,57**
30	1,51	3,1

The bold face numbers represent the significances expected with the level of systematic anticipated by the experimental collaborations (CERN, 2018, p. 109)

However, Alves et al. argue that there is a trade-off between the estimated BDT performance and the efficiency of the cuts. This is due to the observed effect that the various cuts that can be made to eliminate the backgrounds result in a weakening of the correlations and, correspondingly, that as the cuts are loosened the larger number of background events may obscure the signal. Finding joint optimal performance, the authors argue, is “the core of the method presented” (CERN, 2018, p. 109). We can see again here a notion of the importance of the ‘physics intuition’ of the experimental set up playing a role in the constraints on contributions to the model of the measurement process. Rather than assuming improvements to the measurement outcome from the ML algorithms, the authors claim that the “core” insight is in this additional step, which gives a deeper understanding of the model of the measurement process. Whilst the source of improvements is not specific to a detector or the HL-LHC, a capacity for improvement and a space where it is possible is identified. Here we can see a very similar notion to the notion of creativity from the LHC models: creativity as both product and process. The product, an improvement to the model of the measurement process. The process, the iterative process by which the model of the measurement process will be transformed to represent the conditions at the HL-LHC and the insights of the creative product.

Creativity is also projected in the construction of the HL-LHC model of the measurement process in several ways. The first is where those modelling the measurement include the recognised quantified creative contributions, such as from the LHC models, in their extrapolations. The second is where spaces are identified in which creative contributions may be possible in the future. The third is a property of the future collaborations. The identification of spaces where a creative product may be possible, but the process by which it will be obtained remains unspecified, assumes a future epistemic agent will transform the model of the measurement process. We can also see the extended timeline for the dynamic interactions between novelty and value in collaborative creativity. The inclusion of projected creativity in the HL-LHC model is tightly constrained theoretically, experimentally, and practically. The source of these constraints is that the model must be meaningful with regards to the epistemic function of the model: a prediction of capacity of the future measurement process for measurement outcomes.

## Conclusion

This paper began with the surprising observation that creativity is a concept explicitly invoked by members of the ATLAS and CMS collaborations in their descriptions of the conditions for a measurement of the self-coupling of the Higgs. This broad notion was one of being able to do more, i.e. improve with respect to the learning aims of the investigators, from transformations to the model of the measurement process rather than expected improvements such as increases in the available data. Due to the very low predicted signal, in the case study explored, this notion translated to a condition for knowledge production. Whilst model-based accounts of measurement of knowledge have recently been very successful in pointing to the epistemic role of models of measurement processes in accounting for standardisation and the stability of measurements (Mitchell et al., 2017), this case focused on the transformations to the model of the measurement process.

In the first part of the case study, I showed how transformations to a model of the measurement process transformed the measurement outcome and the epistemic value of the measurement outcome. From this example, and the accounts of experimental physicists, a particular notion of creativity was identified. This notion was one of creativity that was quantified through comparisons of improvement attributed to expected or unexpected transformations to the model of the measurement process. This notion of creativity, a quantifiable product, had no threshold or minimum novelty, requiring only a net change across a comparison. However, I showed that this notion of creativity is not understood in the context of single evaluations; instead, the evaluation is ongoing over multiple iterations of the model of the measurement process. Creativity was also a collaborative process, distributed across multiple iterations of models of the measurement process. Importantly, the evaluation of creativity, or the value of novelty, was not only derived from comparisons of measurement outcomes. Recognition of an improved measurement outcome was constrained by recognition of an improved models of the measurement process with respect to the epistemic function of the model, which in the case of the LHC models is to specify what the instruments (physical and symbolic) are measuring. The first part of the case study gives a picture of how creativity can be distributed in a collaborative context: while the lack of a threshold for novelty may seem counter intuitive, and certainly runs counter to many of the notions of creativity previously explored, here we have a picture of the dynamical tension in collaborative creativity. In this picture, there are multiple ongoing novel contributions that are both quantified and constrained, resulting in iterative transformations to the model of the measurement process that increase the epistemic value of the measurement outcome.

The models of the measurement process, in the second part of the case study, have the epistemic function of prediction: prediction of a range of measurement outcomes from the future collaborations, detectors and HL-LHC. Creativity is included in the models of the measurement process in a number of ways. Quantified creativity from the LHC models, the rate of improvement, is projected to predict potential measurement outcomes. Also included in this class of models are future spaces for creativity, spaces where there is the possibility of transformation, but for which the processes by which it will be achieved remain unspecified. Finally, creativity is also included in the models of the measurement processes as a property of the future collaborations, based on the assumption that future epistemic agents will transform the model of the measurement process. For the models of the measurement process to perform the epistemic function of prediction, the models are tightly constrained by the assumed material and epistemic conditions of the future. These predictions represent both the lower limits, and potential reach, of the measurement process, as opposed to a representation of a modelling process to be enacted in the future. The models of the measurement process for the HL-LHC generate knowledge of the capacity of the future measurement apparatus and experimental collaborations for a range of measurement outcomes.

In both parts of the case study, the epistemic function of the model of the measurement process is the conservative force, the higher order conceptual space that forms one part of the concept of creativity in the context of the measurement of the self-coupling of the Higgs. The different epistemic functions of the models constrain how novel contributions are included, or not included, in ongoing iterations of the models of the measurement process. In part one of the case study we saw how this concept of creativity, and this dynamic tension, is distributed across a collaboration. In part two, we saw how this concept and dynamic tension is distributed across time.

## Footnotes

1 Beyond Standard Model physics (BSM) is a catch-all term to describe any physics not described or predicted by the Standard Model of particle physics (SM). Briefly, BSM physics introduces new concepts, particles, or interactions, and is motivated by conceptual problems in the SM; for example, the SM does not include gravity.

2 There was no SM prediction of the mass of a SM Higgs boson; however, predictions were made for the spin and couplings.

3 See Ritson (2020) for more on the potential for contradiction and measurement as an indirect path to new physics. See (Morgan, 2005 and French & Murphy, forthcoming) for more on the potential value of surprising results.

4 Slight modifications were made to the questions asked based on who was being interviewed and their experience.

5 An alternative explanation for the invocation of the concept of creativity by the interviewed experimentalists could of course be that the experimentalists are attempting to gain recognition for their achievements. However, this cynical explanation does not permit for the exploration of both the nature and the significance of the transformations to models of the measurement process explored in this paper.

6 Note that I will only briefly touch on how these models are tested, which is also significant for the recognition of creativity in measurements. Whilst I do not have the space here, Staley has recently shown, also drawing on Tal’s notion of models in measurement, how variation of the parameters of the model is a robustness test (Staley, 2018).

7 Often accounts of creativity just list a number of associated, often ambiguous, concepts rather than providing an outline of how they understand creativity. Klausen has referred to this tradition as “passing the buck” (Klausen, 2010, p. 350).

8 See (Boden, 1994a, 1998, 2004, 2010, 1994b; Currie, 2018a, 2018b; Gaut, 2010; Heinze, 2013; Miettinen, 2006; Simon, 2001; Sternberg & Lubart, 1999).

9 For reviews see (Klausen, 2010; Miettinen, 2006).

10 Conceptual spaces, for Boden, are “structured styles of thought”, which are “any disciplined way of thinking that is familiar to (and valued by) a certain social group” (Boden, 2004, p. 4).

11 The Higgs boson is also predicted to have a quartic coupling (λ_HHHH_), a measurement of which is currently considered experimentally impossible (Plehn & Rauch, 2005).

12 This is similar to the decay of the (intermediate) Higgs into the standard channels (2 photons (γγ), ZZ, WW …..bb). The respective probability for a Higgs to do so is theoretically expressed by the respective coupling, G_hgaga_, g_hzz_, etc.

13 The Z boson is also not directly observable; instead it is inferred from the decay products (electron pairs or muon pairs).

14 See Staley and Beauchemin for a detailed exploration of the challenges of LHC measurements (Beauchemin, 2017; Staley, 2018).

15 See (Franklin, 2013) for more on the significance and historical development of the 3- and 5-sigma as standards for ‘evidence for’ and ‘discovery’ respectively.

16 See, for example, (Giordani & Mari, 2014).

17 There have been analyses published for the following channels in run one: bbbb (ATLAS, 2015b; CMS, 2015), bbγγ (ATLAS, 2015c; CMS, 2016), bbττ (CMS, 2017e), bbττ and γγWW (ATLAS, 2015d); and in run two: bbbb (ATLAS, 2016d, 2018b; CMS, 2018d), bbγγ (ATLAS, 2016b; CMS, 2018c), γγWW (ATLAS, 2016c), bbℓνℓν (CMS, 2018e), bbττ (CMS, 2018b).

18 There are different ways to do this depending on the use of Bayesian or frequentist approaches. See Staley (2018) for a detailed discussion.

19 For example, we could imagine a scenario when an increase in the available data results in less sensitivity to the measurement objectives. Improvements that result in no change to the sensitivity of the measurement outcome would be considered to be ‘creative’ as a result of the comparison, as the expectation in this scenario is that the value of the measurement outcome would have otherwise decreased.

20 There are three broad categories of ML: supervised, unsupervised, and reinforcement learning. See (Wheeler, 2017) for an introductory philosophical text and (Chang, Cohen, & Ostdiek, 2018) for an introductory text on ML in high-energy physics.

21 See (Boge & Grünke, forthcoming) for a detailed discussion of epistemic opacity and high-energy experimental physics.

22 This suggests that the question from philosophers regarding opacity in ML in the context of high-energy physics measurements, has potentially been misdirected. Rather than asking whether ML introduces opacity, the ‘challenge from practice’ is to understand how transparency constrains the introduction of novel techniques and how the robustness of such measurement outcomes is determined.

23 ATLAS will replace the inner tracker, and upgrade parts of the calorimeters, muon systems and trigger (ATLAS, 2017a, 2017b, 2017c, 2017d). CMS will also upgrade parts of the trigger, the calorimeter, and muon systems, and will install a new tracker (CMS, 2017a, 2017b, 2017c, 2017d).

24 CERN Yellow Reports are monographs that contain material that is not for journal publication. The aim is to write for a wide physics audience, and for the monographs to last for some time.
